# Beyond the Transaction: Commitment, Intimacy, and Investment in Online Camming Relationships

**DOI:** 10.1007/s10508-026-03476-4

**Published:** 2026-07-23

**Authors:** Rhonda N. Balzarini, Taylor Himes, Faith Swanson, Brandi Reynolds, Christopher R. Agnew

**Affiliations:** 1 Department of Psychology Main Office, Texas State University, San Marcos, TX 78666 USA; 2 Kinsey Institute, Indiana University, Bloomington, IN USA; 3 Department of Psychological Sciences, Purdue University, Lafayette, IN USA

**Keywords:** Digitally mediated intimacy, Camming, Relationship satisfaction, Intimacy

## Abstract

According to the investment model of commitment processes, relationships are more likely to persist when people are highly satisfied with their partner, invested in the relationship, and perceive poor alternatives to it. Although this model has proven robust across relationship contexts, research has yet to examine commitment processes in online camming relationships—relationships between cam models and members of adult camming websites. An increasing number of people are engaging in online camming relationships, with some reporting that their interactions are not strictly sexual and involve some degree of intimacy. Yet, little is known about the factors that facilitate commitment between cam models and members. In a cross-sectional study of cam models (*N* = 199) and members (*N* = 544) from LiveJasmin, surveyed separately (i.e., not as matched dyads), we tested whether satisfaction, investment, and alternatives were associated with current commitment and future planned investment among cam models and members. Based on past research suggesting that alternatives are not as predictive of commitment in multi-partnered relationships and that camming interactions can feature intimacy, we tested an alternative model that replaces the quality of alternatives with intimacy. Results suggest that, in most cases, when cam models or members on camming sites reported greater satisfaction, investment, and intimacy, they, in turn, reported greater current commitment and intentions to invest in their relationship in the future, whereas the quality of alternatives was not a significant predictor. These findings highlight the importance of intimacy in driving commitment within camming relationships, suggesting that emotional connections play a vital role in sustaining these interactions beyond financial exchanges and relationship satisfaction.

**Supplementary Information:**

The online version contains supplementary material available at 10.1007/s10508-026-03476-4.

## Introduction

Erotic webcam sites (commonly referred to as cam sites) have become a major arena for digitally mediated sexual and social interaction, with millions of users worldwide and thousands of performers online at any given time (Wright et al., 2023; Rabouin, 2016). Unlike pre-recorded pornography, camming involves real-time, interactive exchanges between cam models and paying members, allowing for personalized encounters that can include sexual activity, casual conversation, or companionship. These repeated, synchronous interactions often foster a sense of familiarity and continuity between models and members, and in some cases, participants describe developing genuine emotional bonds or even “real” romantic relationships (Jones, 2020; Milrod & Weitzer, 2012). At the same time, camming is rooted in economic exchange. Members typically pay for access to models’ time and performances, while models often view camming as a primary source of income (Pitts et al., 2004; Tsang, 2018). In this sense, financial motivations for models and sexual or relational needs for members intersect, creating a context in which intimacy is both commodified and, at times, authentically experienced. While some research highlights the centrality of financial motives in digital sex work (Sanders, 2005), other studies show that emotional intimacy and connection frequently emerge alongside economic exchange (Jones, 2020; Milrod & Weitzer, 2012). These findings align with broader research on commodified intimacy in the sex industry, where emotional labor is produced, performed, and exchanged as part of the service. For instance, in the context of the “girlfriend experience,” sex workers purposefully cultivate encounters that include affection, companionship, and conversation—not just sexual activity (Bernstein, 2007; Sanders, 2008). These arrangements challenge conventional boundaries between commercial and personal relationships and suggest that financial exchange does not preclude authentic emotional engagement. Specific exchanges within camming relationships may differ in important ways, however: They are digitally mediated, typically synchronous (but sometimes asynchronous), and shaped by the affordances of specific platforms (e.g., public vs. private chatrooms, tip-based interactions, subscription models).

Within the broader sex industry, camming occupies a distinct position. Like other forms of transactional sex, camming involves an explicit exchange of money for erotic or relational labor. However, unlike in-person sexual services (e.g., escorting or street-based sex work), camming interactions are digitally mediated, geographically unbounded, and typically occur without physical contact. At the same time, camming differs from asynchronous online sexual services (e.g., pre-recorded pornography or subscription-based platforms) in that interactions are live, reciprocal, and often involve repeated interpersonal exchange. These features place camming at the intersection of transactional sex and relational intimacy. Although camming is legal in many jurisdictions, relationships formed on these platforms remain non-normative and stigmatized, reflecting broader moral and social regulation of sex work (Benoit et al., 2018; Sanders, 2005). This combination of economic exchange, sustained interaction, and social stigma makes camming a particularly informative context for examining commitment processes.

Such features also introduce unique relational dynamics that warrant further empirical attention. Broader research on computer-mediated communication (e.g., Baym, 2015) suggests that online relationships can foster meaningful intimacy; however, the processes through which this occurs may differ from those in face-to-face relationships in terms of pacing, cues, and expectations. To date, little is known about the circumstances that promote intimacy and commitment in camming relationships or the conditions under which these relationships extend beyond paid sexual interactions.

Taken together, these dynamics suggest that camming relationships cannot be understood solely as transactional exchanges or purely as romantic ties. Instead, they occupy a hybrid space where intimacy and economic motivations intertwine. This makes them an important testing ground for established relationship theories. In particular, the investment model of commitment processes (Rusbult et al., 2012) provides a useful framework for examining how satisfaction, investment, quality of alternatives, and intimacy may shape commitment in these digitally mediated, financially structured interactions. Applying this framework allows us to ask whether the same mechanisms that sustain commitment in offline romantic partnerships also operate in camming relationships or whether these connections deviate from established patterns in unique ways.

### Commitment Processes in Relationships

According to interdependence theory (Machia et al., 2020; Thibaut & Kelley, 1959), people evaluate their relationships by weighing the perceived rewards and costs of interactions with a partner. When an individual’s beliefs about the rewards derived from a relationship (e.g., pleasure, satisfaction, fulfillment, financial security) exceed their perception of costs (e.g., increased responsibility, distress, anxiety, despair, pain, fear), people are more likely to be satisfied with that relationship. Conversely, when partners perceive that the costs of a relationship exceed its rewards, they are less likely to be satisfied with it. Furthermore, interdependence theory asserts that we compare current relationship outcomes derived from a relationship against what we perceive we might derive elsewhere (e.g., from another partner, from not being in any relationship). The better we perceive our relationship alternatives to be, the less committed we are to a given relationship.

The investment model of commitment processes (Rusbult, 1980, 1983, 2012), which is based on the interdependence theory notions of relationship satisfaction and alternatives, proposes that the motivation to maintain a relationship is also a function of investment size—or the direct and indirect resources (e.g., time invested, cognitive interdependence that has formed, plans for the future, shared experiences) that are connected to a relationship (Agnew et al., 1998; Goodfriend & Agnew, 2008; Rusbult, 1980, 1983, 2012). The greater the investment in a relationship, the greater the commitment to that relationship. The central prediction of the investment model—that commitment will be higher with greater relationship satisfaction, poorer perceived quality of alternatives, and greater investments—has received considerable support from cross-sectional (Rusbult et al., 1986), scenario (Rusbult, 1980), and longitudinal studies (e.g., Impett et al., 2018; Johnson & Rusbult, 1989; Rusbult, 1983; see Le & Agnew, 2003 and Tran et al., 2019 for meta-analytic reviews). Moreover, the investment model has been supported for various types of interpersonal bonds, including same-sex relationships (Duffy & Rusbult, 1986; Greene & Britton, 2015), consensually non-monogamous relationships (Balzarini et al., 2019), friendships (Branje et al., 2007; Vanderdrift et al., 2012), and even commitment to romantic relationships that recently have dissolved (Tan et al., 2015; Tassy & Winstead, 2014).

Although past research strongly supports the investment model in predicting commitment among romantic couples, the vast majority of studies have focused on relationships with some level of face-to-face interaction (e.g., couples who regularly see their partner in person). No prior research has examined commitment processes among online intimate couples, particularly cam model–member relationships, where interactions entail a compensated financial exchange. Investigating investment and commitment processes in this context is especially interesting, as these relationships involve transactional intimacy: Cam models receive payment for their interactions, and members provide compensation in return. Further, there is a lack of research examining commitment processes among couples whose relationships exist exclusively online. Thus, one aim of the current research was to extend past work by examining commitment processes among online intimate cam partners and to investigate factors that predict commitment among cam models and their members.

### Investment in Camming Relationships

The use of cam sites is increasing in popularity. A recent nationally representative study found that 18% of Americans reported engaging with cam sites (Gesselman et al., 2023). Other research underscores the widespread use of these platforms; for example, in 2024, LiveJasmin reported more than nine million unique monthly viewers (Lindner, 2024). The rising popularity of camming is further reflected in online traffic rankings, with LiveJasmin appearing among the top 100 most visited websites globally (Rabouin, 2016). This trend parallels broader consumer interest in adult content, as reflected in the global traffic to sites such as XVideos and Pornhub, which currently rank as the 7th and 10th most visited websites worldwide—surpassing Amazon, TikTok, OpenAI, Netflix, and Zoom (Wright et al., 2023). Unlike these pre-recorded, asynchronous platforms, however, camming sites such as LiveJasmin provide real-time, interactive exchanges that allow for personalized and dynamic interactions between models and users.

Cam sites are also highly profitable. While it is challenging to estimate exact user counts and revenue figures across platforms, recent trends indicate the growing economic impact of online adult content. For instance, although OnlyFans is not strictly a camming platform—because most of its content is asynchronous and creator-controlled—its growth illustrates the rising demand for digitally mediated sexual content. Between 2019 and 2023, its user base expanded from 13.5 million to more than 305 million, and gross revenue increased from $270 million to $6.63 billion (Statista, 2024). One of the primary draws of cam sites, in contrast to platforms like OnlyFans or traditional pornography, is their live, interactive format. These platforms allow for real-time communication between cam models and members, often creating experiences that feel more personal and emotionally engaging (Kaufman et al., 2024; Milrod & Monto, 2025; Our Culture Magazine, 2023).

Given that cam interactions are perceived as more immersive and authentic than pre-recorded videos (Kaufman et al., 2024; Milrod & Monto, 2025), past research has found that many cam site users develop significant relationships with cam models, including deep emotional connections. For example, research has shown that 60% of clients report having a current emotional bond with at least one cam model, 65% report having ever had an emotional bond with a cam model, 64% report that their interactions with cam models at least sometimes fulfill their emotional needs, and 51% report that they feel cam models somewhat care about their lives beyond their commercial exchange (Kaufman et al., 2024). The reasons and motivations among cam models for sex with real-life romantic partners and virtual clients are strikingly similar, with the top two reasons, pleasure and physical desirability of a partner, being identical for both groups (Kelberga & Martinsone, 2023). Love and commitment were the 4th ranked reason for cam models to engage in sex with virtual clients (3rd reason for real-life partners), and obtaining resources was ranked 6th for virtual clients and 11th (out of 16) for romantic partners, indicating that the motivations for cam models and virtual client relationships are very similar to real-life, in-person romantic relationships and possess a relatively high level of emotional involvement (Kelberg & Martinsone, 2023). However, past research has not examined commitment processes among partners in cam model–member relationships.

### The Current Study

The present research sits at the intersection of relationship science, sexuality research, and the growing literature on digitally mediated intimacy. In the current research, we drew on the investment model of commitment processes (Rusbult, 1980; Rusbult et al., 2012) to examine how commitment develops and is maintained in digitally mediated camming relationships between cam models and members on the popular camming site LiveJasmin. Although the investment model has received extensive empirical support across many relationship contexts, commitment processes have rarely been examined in relationships that are fully online and embedded within a commercial platform. Camming relationships, therefore, provide a unique context for testing whether established commitment processes generalize to digitally mediated, monetized forms of intimacy.

Consistent with the investment model, we predicted that relationship satisfaction and investment would be positively associated with commitment. However, we made one key adaptation to the model to reflect the relational dynamics of camming. In this context, interactions occur in the known presence of multiple potential partners, and exclusivity is neither expected nor structurally enforced. As a result, perceived quality of alternatives may be less meaningful than in monogamous, in-person relationships. Instead, prior research suggests that emotional connection, personal disclosure, and felt intimacy are central to engagement in camming relationships. Accordingly, we treated intimacy as the third relational process predicting commitment, rather than the quality of alternatives.

We tested the following preregistered hypotheses:

#### Hypothesis 1

When cam models or members report higher relationship satisfaction, greater investment, and higher intimacy with a given partner, they will report higher levels of commitment to that relationship.

#### Hypothesis 2

Greater commitment will, in turn, be associated with stronger intentions for future investment in the relationship (for members) or expectations of receiving future investment (for cam models).

In addition to these primary hypotheses, we conducted planned analyses examining associations among satisfaction, investment, intimacy, commitment, and future investment, and conducted exploratory analyses examining whether these associations differed by participant role (cam model vs. member). Additional preregistered analyses examining differences by relationship characterization (romantic vs. casual) are reported in Supplemental Materials.

All hypotheses and analytic plans were specified in advance and are available on the Open Science Framework (OSF) prior to data collection and analyses.

## Method

### Participants and Procedure

Data were collected from cam models and paying clients on LiveJasmin’s website. Eligible participants were current adult cam models or paying members of LiveJasmin who were at least 18 years old and fluent in one of the available survey languages. The member survey was offered in English, while the cam model survey was offered in English and translated into Romanian, Russian, and Spanish—languages most commonly spoken by cam models on the platform. To ensure consistency across language versions, we used back-translation procedures to identify and resolve any discrepancies (see Colina et al., 2016; Tyupa, 2011). Paying members were exposed to a pop-up advertisement after spending 10 s on the LiveJasmin ListPage (exclusively). The pop-up ad said, “Help Science! Take a quick survey about LiveJasmin.” To recruit cam models, the same advertisement was included in a newsletter sent out to cam models by LiveJasmin’s Account Manager. Interested cam models and members could click “Start now” on the advertisement to be redirected to a Qualtrics survey, which began with a consent form and then proceeded to survey and demographic questions. Participants were then automatically shown a written debriefing screen within Qualtrics that explained the study purpose and confirmed that their participation had concluded; the same debriefing procedure was used for both cam models and members. The debriefing page also briefly described the researchers’ focus (e.g., commitment processes) and provided references on the investment model and related constructs for interested participants. Importantly, cam models and members were recruited independently, and responses were not linked at the dyadic level. Thus, the data represented individuals’ perceptions of their longest standing camming connection rather than matched model–member pairs. Accordingly, all analyses were conducted separately by participant role and should not be interpreted as capturing both partners’ perspectives within the same relationship.

The total sample included 743 participants. The final sample of cam model participants (*n* = 199) consisted of 169 women, 14 men, and 16 individuals who selected an alternative gender option. Most cam model participants identified as straight or heterosexual (61.8%) and were in their early 30 s (*M* = 30.7, *SD* = 8.27). The final sample of participants who were paying members on LiveJasmin (*n* = 544) included mostly men (512 men, 22 women, and 10 members who selected another gender option). Most member participants identified as straight or heterosexual (91.2%) and were in their mid-40 s (*M* = 44.5, *SD* = 13.82). Table 1 presents additional demographic information.

**Table 1:** Sample demographics

	Models (*n* = 199)	Members (*n* = 544)
	Mean (*SD*) or N (%)	Mean (*SD*) or N (%)
Age (in years)	30.72 (8.27)	44.51 (13.82)
*Gender*		
Man	14 (7.04%)	512 (94.12%)
Woman	169 (84.92%)	22 (4.04%)
Non-binary	5 (2.51%)	2 (0.37%)
Agender	1 (0.5%)	0 (0.0%)
Another identity not listed	9 (4.52%)	1 (0.18%)
Prefer not to answer	1 (0.5%)	2 (0.37%)
Did not answer	0 (0.0%)	5 (0.92%)
*Region of residence*		
North America	8 (4.02%)	275 (50.55%)
South America	83 (41.71%)	2 (0.37%)
Europe	77 (38.69%)	193 (35.48%)
Middle East	0 (0.00%)	13 (2.39%)
Africa	4 (2.01%)	5 (0.92%)
Asia	22 (11.06%)	16 (2.94%)
Oceania	0 (0.00%)	25 (4.60%)
Did not answer	3 (1.51%)	13 (2.39%)
*Sexual orientation*		
Heterosexual	123 (61.81%)	496 (91.18%)
Lesbian	3 (1.51%)	2 (0.37%)
Gay	5 (2.51%)	1 (0.18%)
Bisexual	57 (28.64%)	31 (5.70%)
Asexual	3 (1.51%)	5 (0.92%)
Pansexual	2 (1.01%)	2 (0.37%)
Another sexual orientation	6 (3.02%)	5 (0.92%)
*Relationship status*		
Single and not dating anyone	76 (38.19%)	239 (43.93%)
Single but casually dating	26 (13.07%)	84 (15.44%)
Seriously dating one person	13 (6.53%)	23 (4.23%)
Seriously dating more than one person	0 (0.0%)	7 (1.29%)
In a committed but open relationship^*^	10 (5.03%)	18 (3.31%)
*In a committed, exclusive relationship*		
with one person	64 (32.16%)	136 (25.00%)
Another relationship status	8 (4.02%)	31 (5.70%)
Did not answer	2 (1.01%)	6 (1.10%)
Relationship Length (years)	2.33 (2.82)	2.88 (3.08)

^*^Defined to participants as “(e.g., you and/or your partner have permission to see other people)”

### Measures

**Fig. 1:**
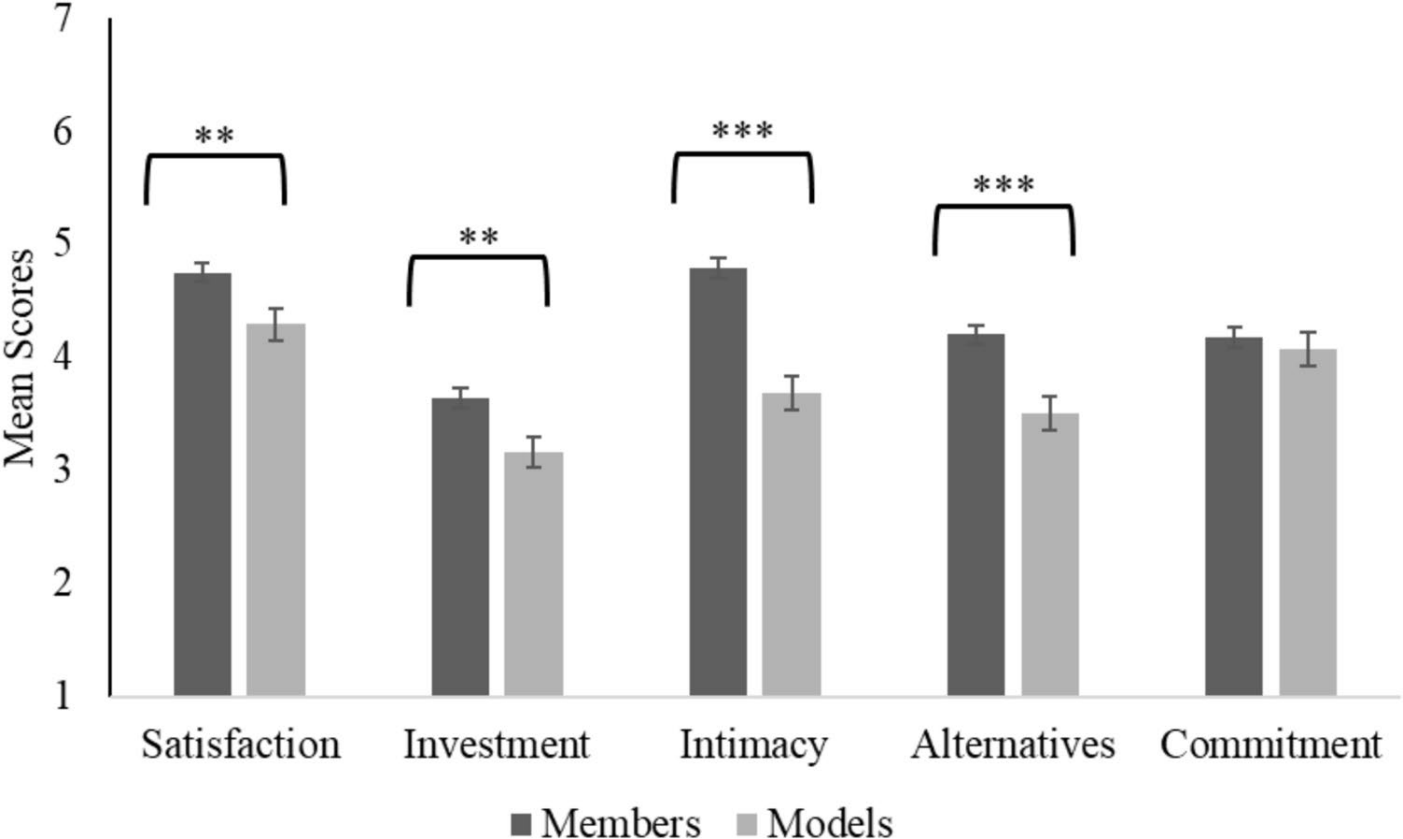
Mean comparisons between members and cam models on focal variables. Note: ***p < .001, **p < .01, *p < .05

To ensure that responses reflected a consistent relational context—rather than a series of brief or disconnected interactions—all participants were first asked whether they were interacting with more than one cam model or member on LiveJasmin. Those who responded “yes” were then instructed to answer all subsequent questions with reference to the individual they had interacted with the longest. This approach allowed us to meaningfully assess constructs such as intimacy and commitment within participants’ most established camming connection. Beyond this anchoring prompt, there was no imposed minimum threshold for interaction (e.g., specific duration, type of session, or monetary investment). Our goal was to capture the full range of relational experiences represented on the platform, from casual interactions to more sustained and emotionally involved connections. Participants then completed truncated versions or single items from validated scales that have been used in past research (e.g., Balzarini et al., 2021, 2023), and selected to reduce survey length and fatigue, increase efficiency, and minimize participant attrition (Bolger et al., 2003). The survey for members and models was identical; however, the wording was modified to reflect the target of interest (e.g., members were asked about their investment in their relationships with cam models, and vice versa). Correlations between variables within each group are reported in Table 2, and means for cam models and members are displayed in Fig. 1.

**Table 2:** Correlations among study variables

	1	2	3	4	5	6
Satisfaction	–	.38***	.40***	.28***	.54***	.23***
Investment	.52***	–	.59***	.08	.75***	.24***
Intimacy	.59***	.42***	–	.10*	.64***	.26***
Alternatives	.44***	.40***	.43***	–	.15**	.07
Commitment	.62***	.53***	.61***	.36***	–	.29***
Future investment	.24*	.12	.20*	.25**	.17*	–

^***^p < .001, **p < .01, *p < .05. Correlations for models are below the diagonal. Correlations for members are above the diagonal

#### Satisfaction

A single item from the Investment Model Scale (IMS, Rusbult et al., 1998) was used to assess relationship satisfaction (e.g., “I feel satisfied with our relationship”). Responses were provided on a 7-point scale (1 = *do not agree at all*, 4 = *agree somewhat*, 7 = *agree completely*), with higher scores indicating greater relationship satisfaction.

#### Investment

A single item from the IMS (Rusbult et al., 1998) was used to assess investment (e.g., “I have put a great deal into our relationship that I would lose if the relationship were to end”). Possible responses were on a 7-point scale (1 = *do not agree at all*, 4 = *agree somewhat*, 7 = *agree completely*), with higher scores indicating greater investment in the relationship.

#### Alternatives

A single item from the IMS (Rusbult et al., 1998) was used to assess the perceived quality of alternatives (e.g., “My alternatives to this member (cam model) are close to ideal (dating another, spending time with friends or on my own, etc.”). Possible responses were provided on a 7-point scale (1 = *do not agree at all*, 4 = *agree somewhat*, 7 = *agree completely*), with higher scores indicating greater quality of alternatives.

#### Intimacy

A single item from the Perceived Relationship Quality Components Scale (PRQC; Fletcher et al., 2000) was used to measure intimacy (e.g., “How intimate is your relationship with this member/cam model?”). Participants were asked to respond on a 7-point scale (1 = *not at all*, 7 = *extremely*), with higher scores indicating greater intimacy between models and members.

#### Commitment

A single item from the IMS (Rusbult et al., 1998) was used to assess commitment (e.g., “I am committed to maintaining my relationship with this member/cam model”). Responses were provided on a 7-point scale (1 = *do not agree at all*, 4 = *agree somewhat*, 7 = *agree completely*), with higher scores indicating greater relationship commitment.

#### Future Member Investment

Future investment by users was assessed with a single-item question, worded as appropriate for cam models and members (“In the future, how much do you intend to pay for interactions with this model in credits?” “In the future, how much do you think this member intends to pay for interaction in credits with you”). On LiveJasmin, credits were the platform’s virtual currency used to interact with cam models and access premium content. Members purchase credits with real money and spend credits for various purposes, such as private shows (i.e., paying for exclusive, one-on-one time with a performer), tips (i.e., sending gratuities to a cam model to show appreciation or encourage specific actions), exclusive content (i.e., unlocking videos, photos, or other media uploaded by performers), or features and interactions. Participants were asked to enter the amount they projected they would spend on credits in the next month.

#### Analytic Approach

The data and syntax for all analyses reported in this paper were made available on the OSF.^1^ Table 2 reports correlations between all study variables, and Fig. 1 displays descriptive information for cam models and members. To assess whether cam models or members differed in their reports of commitment processes, we conducted one-sided *t*-tests to compare mean levels. We examined whether the statistical assumptions for t-tests were met for each analysis. Wilcoxon rank-sum nonparametric test for independent samples was used when assumptions for the *t*-test were violated (Hodges & Lehmann, 1956).

To test our hypotheses, we conducted hierarchical regression analyses with reports of satisfaction, investment, quality of alternatives, and intimacy serving as independent variables predicting reports of commitment. We first tested the standard investment model, regressing satisfaction, investment, and alternatives onto commitment (Model 1). Next, we tested a statistical model substituting intimacy for alternatives (Model 2). Finally, we tested the investment model variables along with intimacy simultaneously (Model 3). Effects for cam members and models were examined separately, and the adjusted R^2^ for each statistical model was compared to determine which provided the optimal fit to the data.

Based on the best-fitting model, we then examined the role of commitment in projections of members’ future financial investment in these relationships by both members and models. Using path analyses, we assessed whether the optimal model of commitment was significantly associated with projections of financial investment.

## Results

### Descriptive Statistics

Participants were asked whether they considered themselves to be in an online intimate relationship with a member or model on LiveJasmin, indicating whether they viewed this relationship as casual or more committed in nature. Among members, 42.5% reported that their interactions with cam models constituted a relationship, while 57.5% viewed them as casual encounters. Similarly, 31.2% of cam models reported that their interactions with members constituted a relationship, whereas 68.8% described them as casual. These variables were examined descriptively to characterize the range of relational experiences in the sample.

We also asked participants to report the number of partners they had interacted with on the LiveJasmin platform, though the specific question varied based on whether they identified as being in an online intimate relationship. Those who did not report being in such a relationship were asked how many members or models they had interacted with on the platform (e.g., via private chat, VIP shows, cam2cam, or messaging). On average, members in this group reported interacting with 13.86 cam models (*SD* = 13.19), whereas cam models reported interacting with 144.40 members (*SD* = 209.41). In contrast, participants who reported being in an online intimate relationship were asked how many such partners they currently had. Among those participants, members reported an average of 3.42 online intimate partners (*SD* = 1.56), and cam models reported an average of 3.24 online intimate partners (*SD* = 1.19). All values reflect data with outliers removed using the interquartile range (IQR) method.

### Examining Mean Differences in Commitment Processes Between Cam Models and Members

In preliminary analyses, we examined whether mean levels of commitment processes differed between members and cam models. Results from *t*-tests suggested that members reported higher levels of relationship satisfaction (*W* = 46,097, *p* = 0.006), investment (*W* = 46,081,* p* = 0.007), quality of alternatives (*W* = 41,433, *p* < .001), and intimacy (*W* = 37,506, *p* < .001) than did cam models. However, there were no differences in members’ and cam models’ reports of commitment (*W* = 51,448, *p* = 0.583). Additionally, cam models reported higher predicted future investment by members than did members (*W* = 46,400, *p* < .001; see Fig. 1).

### Predicting Commitment of Cam Models and Members

To predict commitment, we conducted hierarchical regression to compare the predictive power of three models separately for members and cam models. Model 1, based on the investment model, included satisfaction, investment, and alternatives. Model 2, our hypothesized model, replaced alternatives with intimacy. Model 3, a combined model, included the investment model variables as well as intimacy. To determine which model provided the strongest prediction of commitment, we examined changes in the adjusted R^2^ (Pedhazur, 1982) while also considering model parsimony. Tables 3 and 4 report the results for members and cam models, respectively.

**Table 3:** Hierarchical multiple regression analysis for commitment among members

Model	Predictor	Estimate	*SE*	*t*	*p*	*Adjusted R*^*2*^	*ΔAdjusted R*^*2*^
Model 1	Intercept	0.13	0.18	0.68	.494	.644	
	Satisfaction	0.34	0.03	9.93	< .001		
	Investment	0.63	0.03	23.10	< .001		
	Alternatives	0.03	0.03	0.82	0.414		
Model 2	Intercept	−0.26	0.17	0.12	.494	.675	+ .031
	Satisfaction	0.30	0.03	8.97	< .001		
	Investment	0.52	0.03	16.89	< .001		
	Intimacy	0.24	0.03	7.12	< .001		
Model 3	Intercept	−0.32	0.19	−1.70	.089	.673	−.002
	Satisfaction	0.29	0.03	8.46	< .001		
	Investment	0.52	0.03	16.85	< .001		
	Alternatives	0.02	0.03	0.80	0.426		
	Intimacy	0.24	0.03	7.00	< .001

**Table 4:** Hierarchical multiple regression analysis for commitment among cam models

Model	Predictor	Estimate	*SE*	*t*	*p*	*Adjusted R*^*2*^	*ΔAdjusted R*^*2*^
Model 1	Intercept	0.80	0.29	2.77	.006	.449	
	Satisfaction	0.51	0.07	7.26	< .001		
	Investment	0.29	0.07	4.20	< .001		
	Alternatives	0.05	0.06	0.76	.450		
Model 2	Intercept	0.64	0.27	2.40	.017	.512	+ .062
	Satisfaction	0.33	0.07	4.60	< .001		
	Investment	0.25	0.06	4.01	< .001		
	Intimacy	0.33	0.06	5.24	< .001		
Model 3	Intercept	0.64	0.27	2.33	.021	.514	+ .002
	Satisfaction	0.35	0.07	4.71	< .001		
	Investment	0.25	0.06	3.89	< .001		
	Alternatives	−0.01	0.06	−0.24	0.814		
	Intimacy	0.33	0.06	5.10	< .001		

For members, Model 2 explained the most variance in commitment (*R*^2^ = 0.675; see Table 3), and adding quality of alternatives did not increase *R*^2^. Additionally, in this model, satisfaction (*t* = 8.97, *p* < .001), investment (*t* = 16.89, *p* < .001), and intimacy (*t* = 7.12, *p* < .001) were all significant predictors of commitment. For cam models, although Model 3 explained the most variance in commitment (*R*^2^ = 0.514), alternatives were not a significant predictor of commitment (*B* = −0.01, *p* = .814). Additionally, the increase in *R*^2^ from Model 2 (satisfaction, investment, and intimacy) to Model 3 (adding alternatives) was negligible (.002; see Table 4). Similarly, as with members, models’ reports of satisfaction (*t* = 4.60, *p* < .001), investment (*t* = 4.01, *p* < .001), and intimacy (*t* = 5.24, *p* < .001) were all significant predictors of commitment in Model 2. Thus, for both members and cam models, Model 2 (satisfaction, investment, and intimacy) provided the best and most parsimonious fit for predicting commitment. Across cam models and members, alternatives were not a significant predictor of commitment, and adding alternatives to any of the models did not account for additional variance in commitment. Accordingly, we proceeded with this model for additional analyses examining planned financial investments in these camming relationships.

### Predicting Future Member Investment by Members in Camming

**Fig. 2:**
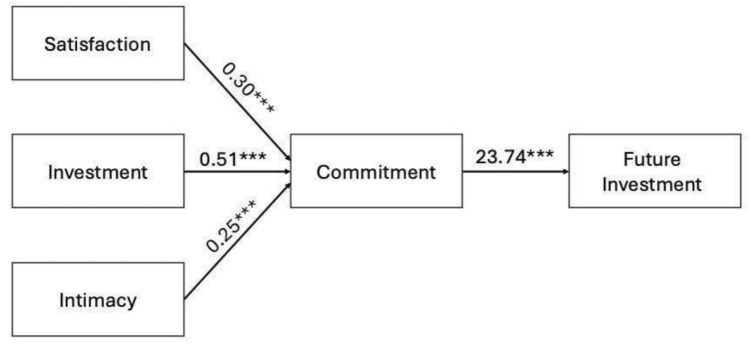
Path analysis of the modified investment model for members. Note: Path coefficients (γ) are standardized estimates from a structural equation model using robust maximum likelihood estimation. ***p < .001, **p < .01, *p < .05

We conducted a path analysis to test whether members’ satisfaction, investment, and intimacy were associated with commitment, and whether commitment, in turn, was associated with anticipated future financial investment (Fig. 2). The model accounted for 65.9% of the variance in commitment and 9.3% of the variance in anticipated future investment. Satisfaction (*γ* = .30, *p* < .001), investment (*γ* = .51, *p* < .001), and intimacy (*γ* = .25, *p* < .001) were each associated with greater commitment, which was in turn associated with higher anticipated future investment (*γ* = 23.74, *p* < .001). Model fit indices were strong (*χ*^2^ = 7.32, *χ*^2^/df = 2.44, CFI = 0.994, TLI = 0.981, RMSEA = 0.057, SRMR = 0.027). Including alternatives did not improve model fit.

**Fig. 3:**
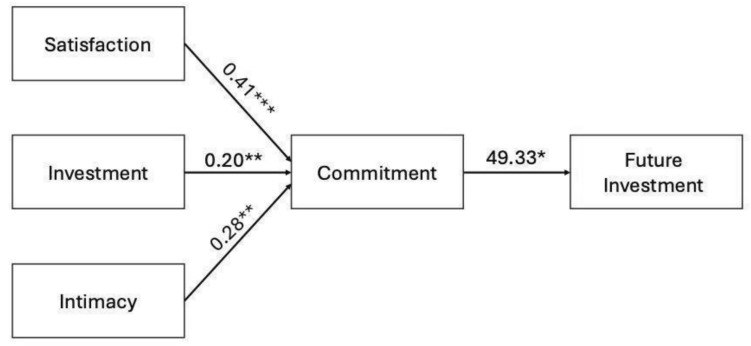
Path analysis of the modified investment model for cam models. Note: Path coefficients (γ) are standardized estimates from a structural equation model using robust maximum likelihood estimation (MLR). ***p < .001, **p < .01, *p < .05

We then estimated the parallel model for cam models (Fig. 3). This analysis accounted for 48.9% of the variance in commitment and 3.0% of the variance in anticipated future investment. Satisfaction (*γ* = .41, *p* < .001), investment (*γ* = .20, *p* = .001), and intimacy (*γ* = .28, *p* = .001) were associated with commitment, which in turn was associated with anticipated member investment (*γ* = 49.33, *p* = .025). Model fit indices again supported the model (*χ*^2^ = 5.87, *χ*^2^/*df* = 1.96, CFI = 0.987, TLI = 0.957, RMSEA = 0.079, SRMR = 0.045).

## Discussion

The present study examined commitment processes within online camming relationships through the lens of the investment model, testing whether satisfaction, investment, alternatives, and intimacy were associated with commitment and, in turn, future investment in the relationship. Given that camming relationships are frequently structured as non-exclusive, we anticipated that alternatives would play a limited role and examined intimacy as an additional predictor of commitment. Results indicated that greater satisfaction, investment, and intimacy were each associated with higher commitment, which in turn was associated with stronger intentions for future investment among both members and cam models, whereas perceived alternatives did not significantly predict commitment.

To our knowledge, this is the first study to empirically compare commitment processes across camming relationships involving both paying members and cam models. Although the present study does not directly compare camming relationships to other forms of commercially mediated intimacy, prior work has documented emotional and relational bonds in a range of non-normative and sex industry contexts (e.g., client–seller relationships, sugar arrangements, and other compensated intimate exchanges). Consistent with this broader literature, our findings suggest that economic exchange does not preclude psychological investment, intimacy, or commitment, while also recognizing that relational structures and norms vary substantially across contexts.

Overall, this research extends prior applications of the investment model by incorporating intimacy and future investment intentions, offering new insight into how commitment processes are associated with both current and anticipated relational contributions in monetized, digitally mediated camming interactions.

### Commitment in Camming Relationships

The results suggest that satisfaction and investment remain central to understanding commitment within camming relationships, consistent with prior applications of the investment model (Rusbult, 1980, 1983; Rusbult et al., 2012). Although the quality of alternatives has traditionally been a strong predictor of commitment in monogamous, in-person relationships (Le & Agnew, 2003), it did not significantly predict commitment in the present context. This aligns with previous research on consensually non-monogamous relationships, where individuals with multiple partners do not necessarily perceive alternatives as a threat to their commitment (Balzarini et al., 2017, 2019). Given that camming relationships are inherently transactional and non-exclusive, users may not conceptualize alternatives in the same way as individuals in monogamous romantic relationships. Instead, they may view cam interactions as unique and irreplaceable experiences that can give rise to intimacy, which may in turn be associated with greater commitment.

Intimacy emerged as a significant predictor of commitment, reinforcing the idea that camming interactions often extend beyond purely transactional exchanges. Past research suggests that emotional connection and authentic engagement are key drivers of sustained camming relationships (Kaufman et al., 2026). The current findings are consistent with this perspective, as higher intimacy was associated with greater commitment, even when accounting for other investment model variables. This pattern suggests that members and cam models who develop a deeper sense of connection may be more likely to maintain their interactions over time.

Equally noteworthy, investment was strongly associated with commitment for members, even more so than satisfaction—a pattern that is less commonly observed in the investment model literature (e.g., Lehmiller & Agnew, 2006). While satisfaction typically plays a more central role in predicting commitment relationship contexts (Le & Agnew et al., 2003; Rusbult et al., 2012), the present findings suggest that the unique features of camming relationships may shift the relative importance of investment. Members may perceive the time, energy, and resources they invest—such as financial spending, platform familiarity, and emotional labor—as especially consequential for their ongoing commitment. Because these investments are often tangible and tracked (e.g., tip history, member status), they may be more cognitively salient than day-to-day fluctuations in satisfaction.

This pattern may also reflect the monetized nature of camming relationships, where one partner pays for time and connection, potentially heightening the relevance of past and anticipated investments in shaping commitment. When a member invests in one relationship connection, this may be associated with reduced capacity or willingness to invest in others, thereby increasing the perceived importance of that relationship. In this way, decisions about where to allocate resources may carry particular weight. Moreover, members may rationalize continued commitment as a way to justify prior investments, a dynamic consistent with sunk cost effects and commitment justification theories (Arriaga & Agnew, 2001; Arriaga et al., 2014; Goodfriend & Agnew, 2008; Rego et al., 2016; Rusbult, 1980).

For cam models, we also found that their commitment was closely associated with their perceptions of future investments from the members they engage with. Anticipated future investment may be linked to greater commitment and continued engagement in these relationships, potentially reinforcing the cycle of mutual involvement (Agnew et al., 1998). The prominence of investment in this context is noteworthy and warrants further research.

### Future Investment by Members in Camming Relationships

The path analytic results further indicated that commitment was associated with projected future investment, such that members who reported higher commitment also reported greater intentions to financially invest in the relationship in the future. This finding highlights the practical implications of commitment in camming dynamics. Although the camming industry is often perceived as purely transactional, the psychological processes underlying these relationships share similarities with traditional romantic bonds. The association between commitment and continued financial investment suggests that camming platforms may potentially benefit from fostering environments that encourage deeper connections between cam models and members.

Although we examined participants’ projections for future financial investment, we did not distinguish between different types of investments—tangible versus intangible—which may be particularly relevant given the role of intimacy in these relationships. According to interdependence theory and the investment model, investments can be tangible (e.g., financial contributions, gifts, time spent in live sessions) or intangible (e.g., emotional energy, self-disclosure, trust), with past research suggesting that intangible investments and planned investments are robust predictors of key relational states and outcomes (Goodfriend & Agnew, 2008). Although the current study focused on planned financial investment, members may also invest emotionally by engaging in deeper, more personal conversations, fostering a sense of closeness with the cam model. Given that intimacy was a significant predictor of commitment, future research could examine whether members who develop strong emotional bonds are also more likely to make non-financial investments, such as offering emotional support, defending cam models in online communities, or providing creative input for content.

Additionally, cam models themselves may make substantial investments in these relationships by tailoring interactions to specific members, remembering details about their time together, or offering personalized experiences. Future studies could examine how reciprocal investment patterns between cam models and members are associated with commitment and long-term engagement. It would also be valuable to investigate how these patterns differ based on the temporal nature of the relationship—for instance, whether members who engage with a single cam model over an extended period invest differently than those who frequently interact with multiple cam models. Overall, these findings suggest that future investment in camming relationships is associated with both members’ and cam models’ commitment, and that these investments may extend beyond financial contributions to include a range of tangible and intangible forms. Understanding how these forms of investment interact with commitment and intimacy will be important for further unpacking the dynamics of these relationships.

### Strengths and Limitations

One major strength of the current research is that we examined commitment processes among both cam models and paying members of one of the most popular camming sites at the time of data collection, LiveJasmin. Much prior work has focused almost exclusively on members’ experiences, often overlooking cam models’ perspectives (e.g., Bleakley, 2014; Jones, 2020; Nayar, 2017). By including both groups, this study offers a more comprehensive account of commitment dynamics within digitally mediated camming relationships.

At the same time, these strengths should be interpreted in light of several important limitations. First, the study was cross-sectional, which precludes causal inferences regarding the directionality of associations among satisfaction, investment, intimacy, commitment, and future investment. Longitudinal designs will be necessary to assess how these processes unfold over time.

Second, although we recruited both cam models and members, participants were recruited independently and could not be linked as model–member dyads. Consequently, the data reflect individuals’ perceptions of a focal camming connection rather than mutual perspectives within the same relationship. This limits inferences about reciprocity, perceptual agreement, and dyadic processes (e.g., whether one partner’s commitment corresponds to the other’s). Given that commitment is inherently relational, the absence of dyadic data is particularly important when interpreting the extent to which these processes are shared versus individually experienced. Future research using dyadic or platform-linked designs would be well positioned to examine how commitment processes operate within matched cam model–member pairs.

Additionally, although the cam model sample (*n* = 199) was sufficient to estimate the hypothesized models and yielded statistically moderate-to-large effects, it remains relatively modest for structural equation modeling. Replication with larger and more diverse samples of cam models will help increase confidence in parameter estimates and model generalizability (Wang & Rhemtulla, 2021). The predominance of heterosexual and gender-conforming participants further limits generalization, underscoring the need for future research with more sexual and gender-diverse samples. Moreover, because camming operates across diverse legal and regulatory contexts, future research should examine how jurisdiction-specific frameworks (e.g., criminalization, platform liability laws) shape participation, disclosure, and relational dynamics among models and members—potentially through cross-national comparisons or designs that explicitly assess perceived legal risk.

Additionally, several core constructs were assessed using single-item or truncated measures, reflecting a deliberate trade-off between measurement depth and participant burden given the recruitment context. Members were recruited via brief pop-up invitations while actively using the platform, and cam models typically engage in compensated, time-sensitive work. Because participation in the present study was voluntary and uncompensated, we prioritized minimizing time burden to encourage participation and reduce attrition across both groups. To this end, we employed a concise survey. This trade-off is common in field-based research conducted in ecologically valid but time-constrained environments. Although all items were drawn from well-validated scales (e.g., Fletcher et al., 2000; Rusbult et al., 1998), single-item measures limit reliability estimation and construct breadth; future research should replicate these findings using multi-item scales when longer assessments are feasible.

Finally, the present study relied exclusively on self-reported data. Although participants are uniquely positioned to report on their subjective experiences of satisfaction, intimacy, and commitment, future research would benefit from integrating self-report measures with platform-derived behavioral data. For example, access to objective indicators such as time spent interacting, financial exchanges, frequency of contact, patterns of exclusivity or concurrency, and longitudinal engagement trajectories would allow for more precise modeling of how behavioral investment and structural features of camming interactions are associated with perceived commitment processes. Combining self-reported perceptions with behavioral and platform-level data would provide a more comprehensive understanding of how commitment develops and is maintained within camming contexts.

### Conclusion

This study is the first to apply the investment model to online camming relationships, offering new insights into the factors associated with commitment in these unique digital interactions. The findings suggest that satisfaction, investment, and intimacy—rather than the quality of alternatives—are associated with commitment, which in turn is associated with both members’ intentions to make future financial investments and cam models’ expectations for members’ future financial investments. Importantly, these findings challenge the assumption that intimacy and economic exchange are mutually exclusive. Rather than functioning as purely transactional interactions, camming relationships can involve meaningful emotional connections alongside instrumental exchange. Our results suggest that commercially scaffolded relationships may still support authentic psychological investment, intimacy, and commitment, complicating deficit-based views of paid intimacy as inherently inauthentic.

As the landscape of online intimacy continues to evolve, understanding the psychological mechanisms underlying digital erotic exchanges will be critical for researchers, industry professionals, and individuals navigating these emerging relational forms, as well as for advancing broader theoretical perspectives on digitally mediated intimacy.

## Supplementary Information

Below is the link to the electronic supplementary material.

Supplementary file1 (DOCX 57 kb)

## Footnotes

1 The data and syntax will remain private until the paper is in press. Please feel free to request access: rbalzarini@txstate.edu.
