# Supplementary material for: Beyond the Transaction: Commitment, Intimacy, and Investment in Online Camming Relationships
Source: Arch Sex Behav. 2026 Jul 23;55(5):2181–94. doi: 10.1007/s10508-026-03476-4 (PMC13427922; doi:10.1007/s10508-026-03476-4)
Supplement: Supplementary file 1 — Supplementary file1 (DOCX 57 kb) [file 10508_2026_3476_MOESM1_ESM.docx]

**Supplemental Materials**

**Supplement 1: Participants’ Characterization of Camming Relationships**

The purpose of these analyses was to examine within-group differences in how camming relationships are characterized by paying members and cam models, analyzed separately by participant role. Specifically, we tested whether participants’ characterization of their longest-standing camming connection as an online intimate (romantic) relationship versus a more casual interaction was associated with differences in reported investment, relationship satisfaction, commitment, intimacy, and perceived quality of alternatives. We hypothesized that, within both groups, participants who characterized their camming relationship as romantic (relative to casual) would report higher levels of (a) investment, (b) relationship satisfaction, (c) commitment, and (d) intimacy, as well as (e) lower perceived quality of alternatives. In addition, we examined whether the Investment Model of Commitment (Rusbult et al., 1998) replicated when analyses were conducted separately for relationships characterized as romantic versus casual. These hypotheses and analytic decisions were preregistered: <https://osf.io/2jzg3/overview>.

**Method**

**Participants and Procedure**

Participants and procedures are described in detail in the main manuscript. Briefly, data were collected from paying members and cam models on LiveJasmin via an online survey. Cam models and members were recruited independently, and responses were not linked at the dyadic level. Accordingly, all analyses were conducted separately by participant role and reflect individuals’ perceptions of their longest-standing camming connection rather than matched model–member pairs.

The analytic sample for these supplementary analyses included 544 paying members and 199 cam models who completed the relevant measures.

**Measures**

All measures are described in the main manuscript. For the present analyses, we focus on participants’ characterization of their longest-standing camming connection as an online intimate relationship (yes/no), as well as single-item assessments of relationship satisfaction, investment, commitment, perceived quality of alternatives drawn from the Investment Model Scale (Rusbult et al., 1998) and a single item assessment of intimacy drawn from the Perceived Relationship Quality Components Scale (Fletcher et al., 2000).

**Analytic Plan**

The goal of these supplementary analyses was to examine whether participants’ assessments of their camming relationships differed as a function of relationship characterization. Separate independent-samples *t* tests were conducted within each participant group (cam models and paying members), comparing those who did versus did not characterize their relationship as an online intimate relationship.

In addition, we re-ran the primary hierarchical regression analyses reported in the main manuscript separately within each relationship characterization group (intimate vs. not intimate) to assess whether the Investment Model of Commitment operated similarly across relationship types. Structural equation modeling analyses were not re-estimated due to limited statistical power within these subgroups.

The study rationale, hypotheses, and analytic approach were preregistered and are available on the [Open Science Framework (OSF)](https://osf.io/e3erm/).

***Results***

Given variability in how participants characterized their interactions on the platform, we conducted supplementary analyses to examine whether key study variables differed as a function of relationship characterization. Specifically, we compared participants who conceptualized their longest-standing camming connection as an online intimate (romantic) relationship versus a more casual interaction. These analyses allowed us to assess whether core commitment processes—satisfaction, investment, perceived quality of alternatives, and intimacy—operated similarly across different types of camming relationships.

We first conducted independent-samples *t* tests comparing mean levels of each variable by relationship characterization (see Tables S2–S3 and Figure S1). Across both paying members and cam models, participants who described their camming connection as romantic reported significantly higher levels of satisfaction, investment, intimacy, and commitment than those who characterized the relationship as casual. Among members, perceived quality of alternatives did not differ as a function of relationship characterization (*p* = .126). In contrast, cam models who identified their longest-standing connection as romantic reported significantly higher perceived quality of alternatives than those who characterized the connection as casual (*p* = .002).

To further evaluate whether relationship characterization moderated the Investment Model processes examined in the main manuscript, we re-estimated the hierarchical regression models separately for participants who characterized their connection as romantic versus casual (see Tables S4–S7). Among members, results were highly consistent across both groups: satisfaction, investment, and intimacy were significant predictors of commitment regardless of relationship characterization, whereas perceived quality of alternatives was not a significant predictor in either subgroup. Among cam models, a similar pattern emerged; however, investment was not a statistically significant predictor of commitment among those who characterized their connection as romantic. Given the relatively small sample size in this subgroup (*n* = 62), this null effect should be interpreted with caution and may reflect limited statistical power rather than a substantive deviation from the broader pattern.

Taken together, these findings suggest that the core processes outlined in the Investment Model of Commitment operate similarly across a range of camming relationships, including those characterized as more casual as well as those viewed as more affectively meaningful.

Table S1

*T-test Analysis for Commitment among Cam Models by How They Conceptualized Their Longest-Standing Connection (Romantic vs. Casual)*

| Factor | Not in a Relationship | | In a Relationship | | *t* | *df* | *p* | Cohens d |
| --- | --- | --- | --- | --- | --- | --- | --- | --- |
|  | *M* | *SD* | *M* | *SD* |  |  |  |  |
| Investment | 2.81 | 1.96 | 3.92 | 1.82 | 3.85 | 127.23 | .001 | .57 |
| Relationship Satisfaction | 3.98 | 2.05 | 4.97 | 1.53 | 3.78 | 155.68 | <.001 | .52 |
| Alternatives | 3.21 | 2.04 | 4.15 | 1.97 | 3.07 | 122.81 | .002 | .46 |
| Intimacy | 3.11 | 2.02 | 4.94 | 1.83 | 6.29 | 130.08 | <.001 | .93 |
| Commitment | 3.69 | 2.15 | 4.89 | 1.81 | 4.04 | 139.03 | <.001 | .58 |

Table S2

*T-test Analysis for Commitment among Cam Members by How They Conceptualized Their Longest-Standing Connection (Romantic vs. Casual)*

| Factor | Not in a Relationship | | In a Relationship | | *t or W* | *df* | *p* | Cohens d | |
| --- | --- | --- | --- | --- | --- | --- | --- | --- | --- |
|  | *M* | *SD* | *M* | *SD* |  |  |  |  |  |
| Investment | 2.71 | 1.83 | 4.89 | 1.87 | *t* = 13.49 | 489.5 | <.001 | 1.17 |  |
| Relationship Satisfaction | 4.33 | 1.77 | 5.31 | 1.56 | *t* = 6.82 | 522.88 | <.001 | .58 |  |
| Alternatives | 4.10 | 1.86 | 4.34 | 1.74 | *t* =1.53 | 507.45 | .126 | - |  |
| Intimacy^A^ | 3.84 | 1.95 | 6.06 | 1.17 | *W* = 58216 | - | <.001 | 1.33 |  |
| Commitment^A^ | 3.19 | 1.94 | 5.46 | 1.53 | *W* = 57629 | - | <.001 | 1.28 |  |

Note: ^A^ indicates that the Wilcoxon rank-sum test was used due to violations of normality and homogeneity of variance assumptions. Effect size (Cohen’s *d*) is estimated using pooled standard deviations.

Table S3

*Hierarchical Multiple Regression Analysis for Commitment among Members In an Online Intimate Relationship with a Model on LiveJasmin*

| Model | Predictor | Estimate | *SE* | t | *p* | *Adjusted R^2^* | *ΔAdjusted R^2^* |
| --- | --- | --- | --- | --- | --- | --- | --- |
|  |  |  |  |  |  |  |  |
| **Model 1** | Intercept | 1.56 | 0.30 | 5.08 | <.001 | .517 |  |
|  | Satisfaction | 0.40 | 0.05 | 8.21 | <.001 |  |  |
|  | Investment | 0.39 | 0.04 | 9.70 | <.001 |  |  |
|  | Alternatives | -0.03 | 0.03 | 0.82 | 0.464 |  |  |
| **Model 2** | Intercept | 0.65 | 0.37 | 1.77 | .079 | .536 | +.019 |
|  | Satisfaction | 0.33 | 0.05 | 6.62 | <.001 |  |  |
|  | Investment | 0.35 | 0.04 | 8.61 | <.001 |  |  |
|  | Intimacy | 0.22 | 0.07 | 3.10 | .002 |  |  |
| **Model 3** | Intercept | 0.75 | 0.40 | 1.87 | .062 | .534 | -.002 |
|  | Satisfaction | 0.34 | 0.05 | 6.61 | <.001 |  |  |
|  | Investment | 0.35 | 0.04 | 8.52 | <.001 |  |  |
|  | Alternatives | -0.02 | 0.04 | -0.70 | 0.487 |  |  |
|  | Intimacy | 0.21 | 0.07 | 3.05 | .002 |  |  |

Table S4

*Hierarchical Multiple Regression Analysis for Commitment among Members Not in an Online Intimate Relationship with a Model on LiveJasmin*

| Model | Predictor | Estimate | *SE* | t | *p* | *Adjusted R^2^* | *ΔAdjusted R^2^* |
| --- | --- | --- | --- | --- | --- | --- | --- |
|  |  |  |  |  |  |  |  |
| **Model 1** | Intercept | -0.07 | 0.02 | -0.32 | .748 | .569 |  |
|  | Satisfaction | 0.28 | 0.04 | 6.25 | <.001 |  |  |
|  | Investment | 0.67 | 0.04 | 16.32 | <.001 |  |  |
|  | Alternatives | 0.06 | 0.04 | 1.42 | 0.156 |  |  |
| **Model 2** | Intercept | -0.21 | 0.22 | -1.00 | .319 | .584 | +.031 |
|  | Satisfaction | 0.28 | 0.04 | 6.48 | <.001 |  |  |
|  | Investment | 0.60 | 0.05 | 13.38 | <.001 |  |  |
|  | Intimacy | 0.15 | 0.04 | 3.59 | <.001 |  |  |
| **Model 3** | Intercept | -0.35 | 0.24 | -1.45 | .148 | .583 | -.001 |
|  | Satisfaction | 0.26 | 0.04 | 5.73 | <.001 |  |  |
|  | Investment | 0.60 | 0.05 | 13.34 | <.001 |  |  |
|  | Alternatives | 0.06 | 0.04 | 1.41 | 0.161 |  |  |
|  | Intimacy | 0.15 | 0.03 | 3.49 | <.001 |  |  |

Table S5

*Hierarchical Multiple Regression Analysis for Commitment among Cam Models in an Online Intimate Relationship with a Member on LiveJasmin*

| Model | Predictor | Estimate | *SE* | t | *p* | *Adjusted R^2^* | *ΔAdjusted R^2^* |
| --- | --- | --- | --- | --- | --- | --- | --- |
|  |  |  |  |  |  |  |  |
| **Model 1** | Intercept | 0.80 | 0.64 | 1.27 | .211 | .414 |  |
|  | Satisfaction | 0.51 | 0.13 | 4.45 | <.001 |  |  |
|  | Investment | 0.23 | 0.12 | 1.94 | .057 |  |  |
|  | Alternatives | 0.07 | 0.10 | 0.63 | .531 |  |  |
| **Model 2** | Intercept | 0.59 | 0.61 | 0.96 | .343 | .457 | +.043 |
|  | Satisfaction | 0.44 | 0.14 | 3.03 | .004 |  |  |
|  | Investment | 0.21 | 0.11 | 1.93 | .058 |  |  |
|  | Intimacy | 0.26 | 0.12 | 2.23 | .030 |  |  |
| **Model 3** | Intercept | 0.58 | 0.62 | 0.94 | .354 | .447 | -.010 |
|  | Satisfaction | 0.44 | 0.15 | 3.01 | .004 |  |  |
|  | Investment | 0.21 | 0.11 | 1.81 | .076 |  |  |
|  | Alternatives | 0.00 | 0.11 | 0.00 | 0.998 |  |  |
|  | Intimacy | 0.26 | 0.13 | 2.11 | .039 |  |  |

Note: *N* = 62. Results should be interpreted with caution due to limited statistical power.

Table S6

*Hierarchical Multiple Regression Analysis for Commitment among Cam Models Not in an Online Intimate Relationship with a Member on LiveJasmin*

| Model | Predictor | Estimate | *SE* | t | *p* | *Adjusted R^2^* | *ΔAdjusted R^2^* |
| --- | --- | --- | --- | --- | --- | --- | --- |
|  |  |  |  |  |  |  |  |
| **Model 1** | Intercept | 0.87 | 0.34 | 2.56 | .012 | .403 |  |
|  | Satisfaction | 0.48 | 0.09 | 5.58 | <.001 |  |  |
|  | Investment | 0.29 | 0.09 | 3.34 | .001 |  |  |
|  | Alternatives | 0.03 | 0.08 | 0.40 | .687 |  |  |
| **Model 2** | Intercept | 0.63 | 0.31 | 2.02 | .046 | .472 | +.069 |
|  | Satisfaction | 0.30 | 0.09 | 3.48 | .001 |  |  |
|  | Investment | 0.28 | 0.08 | 3.47 | .001 |  |  |
|  | Intimacy | 0.35 | 0.08 | 4.36 | <.001 |  |  |
| **Model 3** | Intercept | 0.62 | 0.33 | 1.90 | .059 | .474 | +.002 |
|  | Satisfaction | 0.32 | 0.09 | 3.55 | .001 |  |  |
|  | Investment | 0.27 | 0.08 | 3.37 | .001 |  |  |
|  | Alternatives | -0.01 | 0.08 | -0.13 | .896 |  |  |
|  | Intimacy | 0.35 | 0.08 | 4.29 | <.001 |  |  |

Figure S1. Mean Comparison between Members and Cam Models based on Relationship Status


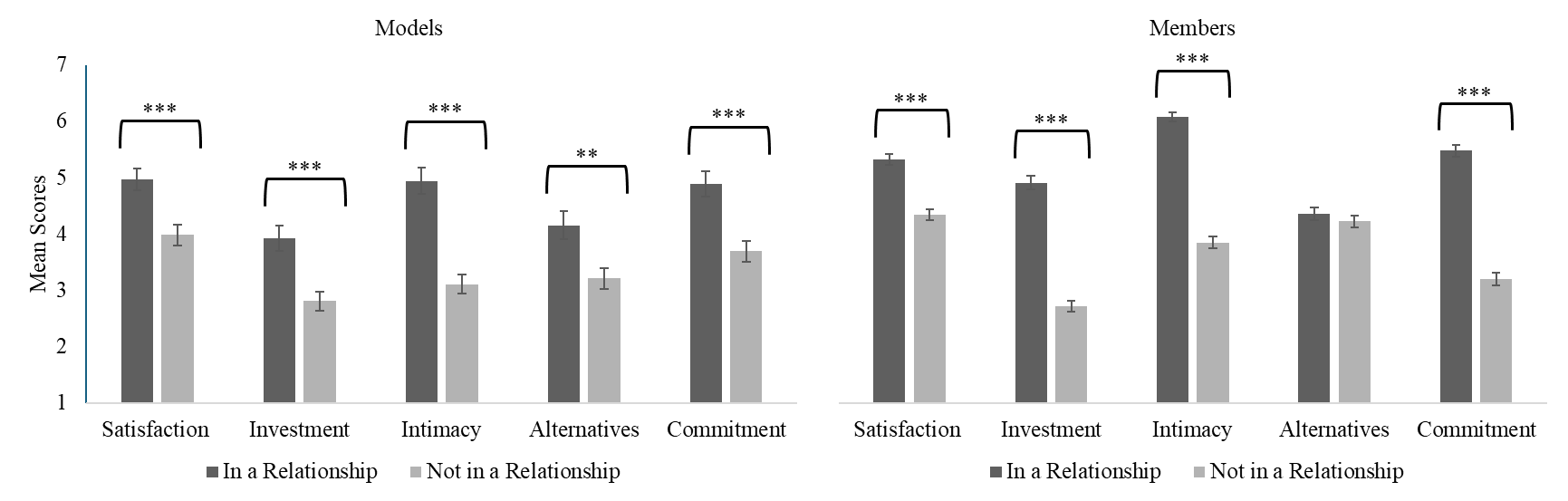


*Note.* The comparisons shown represent dynamics within member–model online intimate relationships only; they should not be interpreted as referring to participants’ offline relationship status or length.

**Supplement 2.** **References for Supplemental Materials**

Fletcher, G. J. O., Simpson, J. A., & Thomas, G. (2000). Perceived Relationship Quality

Components Inventory (PRQC) [Database record]. APA PsycTests.

<https://psycnet.apa.org/doi/10.1037/t03598-000>

Rusbult, C. E., Martz, J. M., & Agnew, C. R. (1998). The Investment Model Scale: Measuring

commitment level, satisfaction level, quality of alternatives, and investment size. *Personal Relationships, 5*, 357–391. <https://psycnet.apa.org/doi/10.1111/j.1475-6811.1998.tb00177.x>
